# Nutritional therapies for mental disorders

**DOI:** 10.1186/1475-2891-7-2

**Published:** 2008-01-21

**Authors:** Shaheen E Lakhan, Karen F Vieira

**Affiliations:** 1 Global Neuroscience Initiative Foundation, Los Angeles, CA, USA

## Abstract

According to the Diagnostic and Statistical Manual of Mental Disorders, 4 out of the 10 leading causes of disability in the US and other developed countries are mental disorders. Major depression, bipolar disorder, schizophrenia, and obsessive compulsive disorder (OCD) are among the most common mental disorders that currently plague numerous countries and have varying incidence rates from 26 percent in America to 4 percent in China. Though some of this difference may be attributable to the manner in which individual healthcare providers diagnose mental disorders, this noticeable distribution can be also explained by studies which show that a lack of certain dietary nutrients contribute to the development of mental disorders. Notably, essential vitamins, minerals, and omega-3 fatty acids are often deficient in the general population in America and other developed countries; and are exceptionally deficient in patients suffering from mental disorders. Studies have shown that daily supplements of vital nutrients often effectively reduce patients' symptoms. Supplements that contain amino acids also reduce symptoms, because they are converted to neurotransmitters that alleviate depression and other mental disorders. Based on emerging scientific evidence, this form of nutritional supplement treatment may be appropriate for controlling major depression, bipolar disorder, schizophrenia and anxiety disorders, eating disorders, attention deficit disorder/attention deficit hyperactivity disorder (ADD/ADHD), addiction, and autism. The aim of this manuscript is to emphasize which dietary supplements can aid the treatment of the four most common mental disorders currently affecting America and other developed countries: major depression, bipolar disorder, schizophrenia, and obsessive compulsive disorder (OCD).

Most antidepressants and other prescription drugs cause severe side effects, which usually discourage patients from taking their medications. Such noncompliant patients who have mental disorders are at a higher risk for committing suicide or being institutionalized. One way for psychiatrists to overcome this noncompliance is to educate themselves about alternative or complementary nutritional treatments. Although in the cases of certain nutrients, further research needs to be done to determine the best recommended doses of most nutritional supplements, psychiatrists can recommend doses of dietary supplements based on previous and current efficacious studies and then adjust the doses based on the results obtained.

## Introduction

Currently, approximately 1 in 4 adult Americans have been diagnosed with a mental disorder, which translates into about 58 million affected people [1]. Though the incidence of mental disorders is higher in America than in other countries, a World Health Organization study of 14 countries reported a worldwide prevalence of mental disorders between 4.3 percent and 26.4 percent [2]. In addition, mental disorders are among the leading causes for disability in the US as well as other countries. Common mental health disorders include mood disorders, anxiety disorders such as post-traumatic stress disorder (PTSD), panic disorders, eating disorders, attention deficit disorder/attention deficit hyperactivity disorder (ADD/ADHD), and autism. However, the four most common mental disorders that cause disabilities are major depression, bipolar disorder, schizophrenia, and obsessive compulsive disorder (OCD) [3,4].

Typically, most of these disorders are treated with prescription drugs, but many of these prescribed drugs cause unwanted side effects. For example, lithium is usually prescribed for bipolar disorder, but the high-doses of lithium that are normally prescribed causes side effects that include: a dulled personality, reduced emotions, memory loss, tremors, or weight gain [5,6]. These side effects can be so severe and unpleasant that many patients become noncompliant and, in cases of severe drug toxicity, the situation can become life threatening.

Researchers have observed that the prevalence of mental health disorders has increased in developed countries in correlation with the deterioration of the Western diet [7]. Previous research has shown nutritional deficiencies that correlate with some mental disorders [8,9]. The most common nutritional deficiencies seen in mental disorder patients are of omega-3 fatty acids, B vitamins, minerals, and amino acids that are precursors to neurotransmitters [10-16]. Compelling population studies link high fish consumption to a low incidence of mental disorders; this lower incidence rate has proven to be a direct result of omega-3 fatty acid intake [10,17,18]. One to two grams of omega-3 fatty acids taken daily is the generally accepted dose for healthy individuals, but for patients with mental disorders, up to 9.6 g has been shown to be safe and efficacious [19-21]. Western diets are usually also lacking in fruits and vegetables, which further contributes to vitamin and mineral deficiencies.

This article will focus on the nutritional deficiencies that are associated with mental disorders and will outline how dietary supplements can be implemented in the treatment of several disorders (see Table 1 for an overview). The mental disorders and treatments covered in this review do not include the broad and complex range of disorders, but however focuses on the four most common disorders in order to emphasize the alternative or complementary nutritional options that health care providers can recommend to their patients.

**Table 1:** Summary of proposed causes and treatments for common mental health disorders

**Mental Disorder**	**Proposed Cause**	**Treatment**	**References**	**Type of Study**
Major Depression	Serotonin deficiency	Tryptophan	[15] [32]	Human pilot clinical trial Double-blind, placebo controlled
	Dopamine/Noradrenaline deficiency	Tyrosine	[30] [36]	Double-blind, placebo controlled Randomized within or between subjects
	GABA deficiency	GABA	[29]	Clinical trial
	Omega-3 deficiency	Omega-3s	[39]	Clinical trial
	Folate/Vitamin B deficiency	Folate/Vitamin B	[9] [13]	Randomized controlled trial Clinical trial
	Magnesium deficiency	Magnesium	[14]	Cases studies
	SAM deficiency	SAM	[37]	Double-blind, placebo controlled

Bipolar Disorder	Excess acetylcholine receptors	Lithium orotate & taurine	[50]	Clinical trial
	Excess vanadium	Vitamin C	[45]	Double-blind, placebo controlled
	Vitamin B/Folate deficiency	Vitamin B/Folate	[47] [71]	Human pilot clinical trial Clinical trial
	L-Tryptophan deficiency	L-Tryptophan	[72]	Clinical trial
	Choline deficiency	Lecithin	[73]	Double-blind, placebo controlled
	Omega-3 deficiency	Omega-3s	[21] [48] [74] [75]	Double-blind, placebo controlled Clinical trial Clinical trial Double-blind, placebo controlled

Schizophrenia	Impaired serotonin synthesis	Tryptophan	[53]	Open-baseline controlled trial
	Glycine deficiency	Glycine	[54] [55] [56]	Double-blind, placebo controlled Human pilot open-label trial Clinical trial
	Omega-3 deficiencies	Omega-3s	[59] [60] [65]	Double-blind, placebo controlled Randomized, placebo controlled Open-label clinical trial

Obsessive Compulsive Disorder	St. John's wort deficiency	St John's wort	[69] [70]	Randomized, double-blind trial Double-blind, placebo controlled

### Major Depression

Major depression is a disorder that presents with symptoms such as decreased mood, increased sadness and anxiety, a loss of appetite, and a loss of interest in pleasurable activities, to name a few [22]. If this disorder is not properly treated it can become disabling or fatal. Patients who are suffering from major depression have a high risk for committing suicide so they are usually treated with psychotherapy and/or antidepressants [23]. Depression has for some time now been known to be associated with deficiencies in neurotransmitters such as serotonin, dopamine, noradrenaline, and GABA [22-27]. As reported in several studies, the amino acids tryptophan, tyrosine, phenylalanine, and methionine are often helpful in treating many mood disorders, including depression [28-33]. Tryptophan is a precursor to serotonin and is usually converted to serotonin when taken alone on an empty stomach. Therefore, tryptophan can induce sleep and tranquility and in cases of serotonin deficiencies, restore serotonin levels leading to diminished depression [15,31].

Tyrosine is not an essential amino acid, because it can be made from the amino acid phenylalanine. Tyrosine and sometimes its precursor phenylalanine are converted into dopamine and norepinephrine [34]. Dietary supplements that contain tyrosine and/or phenylalanine lead to alertness and arousal. Methionine combines with ATP to produce S-adenosylmethionine (SAM), which facilitates the production of neurotransmitters in the brain [35-38]. Currently, more studies involving these neurochemicals are needed which exhibit the daily supplemental doses that should be consumed in order to achieve antidepressant effects.

Since the consumption of omega-3 fatty acids from fish and other sources has declined in most populations, the incidence of major depression has increased [10]. Several mechanisms of action may explain how eicosapentaenoic acid (EPA) which the body converts into docosahexaenoic acid (DHA), the two omega-3 fatty acids found in fish oil, elicit antidepressant effects in humans. Most of the proposed mechanisms involve neurotransmitters and, of course, some have more supporting data than others. For example, antidepressant effects may be due to EPA being converted into prostaglandins, leukotrienes, and other chemicals the brain needs. Other theories state that EPA and DHA affect signal transduction in brain cells by activating peroxisomal proliferator-activated receptors (PPARs), inhibiting G-proteins and protein kinase C, as well as calcium, sodium, and potassium ion channels. No matter which mechanism(s) prove to be true, epidemiological data and clinical studies already show that omega-3 fatty acids can effectively treat depression [39]. Consuming omega-3 fatty acid dietary supplements that contain 1.5 to 2 g of EPA per day have been shown to stimulate mood elevation in depressed patients. However, doses of omega-3 higher than 3 g do not present better effects than placebos and may not be suitable for some patients, such as those taking anti-clotting drugs [40].

In addition to omega-3 fatty acids, vitamin B (e.g., folate), and magnesium deficiencies have been linked to depression [9,13,14]. Randomized, controlled trials that involve folate and B12 suggest that patients treated with 0.8 mg of folic acid/day or 0.4 mg of vitamin B12/day will exhibit decreased depression symptoms [9]. In addition, the results of several case studies where patients were treated with 125 to 300 mg of magnesium (as glycinate or taurinate) with each meal and at bedtime led to rapid recovery from major depression in less than seven days for most of the patients [14].

### Bipolar Disorder

A patient suffering from major depression may also present symptoms such as recurring episodes of debilitating depression, uncontrollable mania, hypomania, or a mixed state (a manic and depressive episode) which is clinically diagnosed as bipolar disorder [41]. Some biochemical abnormalities in people with bipolar disorder include oversensitivity to acetylcholine, excess vanadium, vitamin B deficiencies, a taurine deficiency, anemia, omega-3 fatty acid deficiencies, and vitamin C deficiency.

Bipolar patients tend to have excess acetylcholine receptors, which is a major cause of depression and mania [42,43]. Bipolar patients also produce elevated levels of vanadium, which causes mania, depression, and melancholy [44,45]. However, vitamin C has been shown to protect the body from the damage caused by excess vanadium. A double-blind, placebo controlled study that involved controlling elevated vanadium levels showed that a single 3 g dose of vitamin C decreases manic symptoms in comparison to placebo [45].

Taurine is an amino acid made in the liver from cysteine that is known to play a role in the brain by eliciting a calming effect. A deficiency of this amino acid may increase a bipolar patient's manic episodes. In addition, eighty percent of bipolar sufferers have some vitamin B deficiencies (often accompanied by anemia) [46]. The combination of essential vitamin supplements with the body's natural supply of lithium reduces depressive and manic symptoms of patients suffering from bipolar disorder [47].

Another well-known factor for mental disorders is that cells within the brain require omega-3 oils in order to be able to transmit signals that enable proper thinking, moods, and emotions. However, omega-3 oils are often present at very low levels in most Americans and bipolar sufferers [48]. Numerous clinical trials, including double-blind, placebo controlled studies have been performed which show that 1 to 2 grams of omega-3 fatty acids in the form of EPA added to one's daily intake decreases manic/depressive symptoms better than placebo (See Table 1).

Prescription lithium is in the form of lithium carbonate, and doses can be as high as 180 mg. It is these high doses that are responsible for most of lithium's adverse side effects. Some of the more common side effects include a dulled personality, reduced emotions, memory loss, tremors, or weight gain [5,6]. Another form of lithium called lithium orotate, is preferred because the orotate ion crosses the blood-brain barrier more easily than the carbonate ion of lithium carbonate. Therefore, lithium orotate can be used in much lower doses (e.g. 5 mg) with remarkable results and no side effects [49,50]. Clinical trials involving 150 mg daily doses of lithium orotate administered 4 to 5 times a week, showed a reduction of manic and depressive symptoms in bipolar patients [50]. In addition, lithium orotate is available without a prescription, unlike lithium carbonate, which is considered a prescription drug by the Food and Drug Administration (FDA). Studies have also shown that the amino acid-derivative, taurine, as an alternative to lithium, blocks the effects of excess acetylcholine that contributes to bipolar disorder [51].

Numerous studies for bipolar disorder have been published that list specific lifestyle changes as well as amounts of dietary supplements that can be used to treat this disorder. A summary of these results is listed in Table 2.

**Table 2:** List of possible causes and treatments for bipolar disorder including specific doses as well as supplementary information

**Mental Disorder**	**Proposed Cause**	**Treatment**	**References**
Bipolar Disorder	Food allergies	Avoid foods that elicit an allergic response	[76, 77]
	Caffeine	Avoid coffee and other caffeinated beverages	[78]
	Inhibition of lithium from alkalizing agents	Avoid alkalizing agents like bicarbonates	[79]
	Vitamin B6 deficiency	100–200 milligrams/day	[72, 80]
	Vitamin B12 deficiency	300–600 mcirograms/day	[71, 81–83]
	Vitamin C deficiency	1–3 grams taken as divided doses	[84–86]
	Folate deficiency	200 micrograms/day	[9, 13, 71, 82, 83, 87, 88]
	Choline deficiency	10–30 grams of phosphatidyl form in divided doses	[73, 89]
	Omega-3 or -6 deficiency	500–1000 milligrams/day	[10, 11, 21, 39, 74, 75, 90–94]
	Phenylalanine deficiency	Initially 500 milligrams/day; can increase to 3–4 grams/day	[95, 96]
	Tryptophan deficiency	50–200 milligrams taken as divided doses	[97–100]
	S-Adenosyl-L-Methionine (SAM) deficiency	800 milligrams	[101–103]
	Melatonin deficiency	3–6 milligrams at 9 pm	[104–106]
	Phosphatidylserine deficiency	100 milligrams with food	[107]

### Schizophrenia

Schizophrenia is a mental disorder that disrupts a person's normal perception of reality. Schizophrenic patients usually suffer from hallucinations, paranoia, delusions, and speech/thinking impairments. These symptoms are typically presented during adolescence [52]. Disturbances in amino acid metabolism have been implicated in the pathophysiology of schizophrenia. Specifically, an impaired synthesis of serotonin in the central nervous system has been found in schizophrenic patients [53]. High doses (30 g) of glycine have been shown to reduce the more subtle symptoms of schizophrenia, such as social withdrawal, emotional flatness, and apathy, which do not respond to most of the existing medications [54-56]. An open-label clinical trial performed in 1996 revealed that 60 g of glycine per day (0.8 g/kg) could be given to schizophrenic patients without producing adverse side effects and that this dose led to a two-fold increase in cerebrospinal fluid (CSF) glycine levels [55]. A second clinical study treated patients with the same dosage divided into 3 doses within 1 week. This form of glycine treatment led to an eight-fold increase in CSF glycine levels [56].

The most consistent correlation found in one study that involved the ecological analysis of schizophrenia and diet concluded that increased consumption of refined sugar results in an overall decreased state of mind for schizophrenic patients, as measured by both the number of days spent in the hospital and poor social functioning [57]. That study also concluded that the dietary predictors of the outcome of schizophrenia and prevalence of depression are similar to those that predict illnesses such as coronary heart disease and diabetes.

A Danish study showed that better prognoses for schizophrenic patients strongly correlate with living in a country where there is a high consumption of omega-3 fatty acids [58]. Eicosapentaenoic acid (EPA), which is found in omega-3 fish oils, has been shown to help depressive patients and can also be used to treat schizophrenia [41,42,59]. Furthermore, studies suggest that supplements such as the commercially available VegEPA capsule, when taken on a daily basis, helps healthy individuals and schizophrenic patients maintain a balanced mood and improves blood circulation [59-65].

The VegEPA capsule contains:

• 280 milligrams of EPA from marine omega-3 fish oil

• 100 milligrams of organic virgin evening primrose omega-6 oil

• 1 milligram of the anti-oxidant vitamin E

• An outer capsule made out of fish gelatine

For schizophrenic patients, docosahexaenoic acid (DHA) supplements inhibit the effects of EPA supplements so it is recommended that the patient only takes the EPA supplement, which the body will convert into the amount DHA it needs [59-65]. Double-blind, placebo controlled studies, randomized, placebo controlled studies, and open-label clinical studies have all shown that approximately 2 g of EPA taken daily in addition to one's existing medication effectively decreases symptoms in schizophrenic patients [59,60,65].

### Obsessive-Compulsive Disorder

Obsessive compulsive disorder (OCD) is an anxiety disorder that causes recurring stressful thoughts or obsessions that are followed by compulsions, which are repeated in an uncontrollable manner as a means of repressing the stressful thought [66]. It is well documented that selective serotonin reuptake inhibitors (SSRIs) help patients with OCD [67]. Therefore, it is clear that nutrients which increase serotonin levels will reduce the symptoms of OCD. As discussed earlier, the amino acid tryptophan is a precursor to serotonin, and tryptophan supplements (which are better than 5-Hydroxytryptophan) will increase serotonin levels and treat OCD [68].

A commercially available supplement called Amoryn has recently proven to help patients suffering from depression, anxiety, and OCD [69,70]. The main ingredient in Amoryn, St. John's wort, has been shown to help OCD patients better deal with their recurring thoughts and compulsions. Two double-blind, placebo-controlled studies were recently performed that compared the affects of a 900 mg daily dose of St. John's wort extract to 20 mg daily doses of Paroxetine (Paxil) or Fluoxetine; which are both SSRIs used to treat OCD. In comparison to patients taking Paxil, those who took the St. John's wort supplement showed a 57% decrease in OCD symptoms and were 47% less likely to exhibit side effects [69]. In comparison to patients taking Fluoxetine, consumption of the St. John's wort extract reduced 48% of OCD patient's symptoms [70]. These results clearly depict how the use nutritional supplements can be effective treatments for mental disorders.

## Conclusion

Here we have shown just a few of the many documented nutritional therapies that can be utilized when treating mental disorders. Many of these studies were done in the 1970s and 1980s, but were soon discontinued because they were underfunded. Nutritional therapies have now become a long-forgotten method of treatment, because they were of no interest to pharmaceutical companies that could not patent or own them. Instead, the companies that funded most clinical research spent their dollars investigating synthetic drugs they could patent and sell; these drugs however usually caused adverse side effects.

There is tremendous resistance to using supplements as treatments from clinicians, mostly due to their lack of knowledge on the subject. Others rather use prescription drugs that the drug companies and the FDA researches, monitors and recalls if necessary. However, for some patients, prescription drugs do not have the efficacy of nutritional supplements and they sometimes have far more dangerous side effects. So for clinicians to avoid these supplement therapies because of a lack of knowledge and unwillingness to use treatments not backed by drug companies and the FDA, they are compromising their patients' recovery due to their own laziness or selfishness.

Clinical studies that show the ability of a prescription drug to effectively treat mental disorders will often argue that supplements as treatments, when unmonitored, are more risky than prescription drugs and may ineffectively treat a patient's symptoms. For example one study listed several methods of treatment, none of which include natural compounds, for OCD patients that include: megadoses of SSRIs, intravenous chlomipramine, oral morphine, deep brain stimulation, and functional neurosurgery [67]. Most of these treatments are invasive or unnatural and will inevitably cause severe side effects to the patient, whose symptoms will probably still reoccur over time. Another example of the literature scaring clinicians away from supplement therapies is an article that warns patients about the dangers of consuming high amounts of omega-3 fatty acids. This manuscript involves a patient who was taking approximately 10 times more than the recommended dose of omega-3 supplements [40]. Numerous studies have shown that up 2 grams of EPA (omega-3 fatty acid) taken daily is sufficient for decreasing symptoms of several mental health disorders with no side effects. This publication with a megadose of omega-3 fatty acids stresses the importance of monitoring the consumption of supplements as well as prescribed drugs, preferably through regular consultations with a licensed health care professional.

Proper medical diagnosis and a clear description of all possible treatment options should always be the first plan of action when treating mental disorders. However, the final decision on whether or not to try nutritional supplements as a treatment must be based on the patient preferences. Now with consumers becoming more interested in natural and holistic therapies, nutritional therapies have been well-received, and some studies are again underway in these areas. New well-designed clinical studies are being published daily on the positive effects of nutritional and supplement therapies on all types of disorders and diseases. It will take some time for clinicians to become educated on all the options available, but this is an important task that should not be ignored.

Those with influence in this field should continue to examine natural treatments on the scientific level in order to increase the availability of grant money for this type of research. This will lead to a surge of researchers who will submit proposals for grants enabling laboratories to further investigate the hypothesis that proper nutrition contributes to better mental health.

Psychiatrists treating patients with mental disorders should be aware of available nutritional therapies, appropriate doses, and possible side effects in order to provide alternative and complementary treatments for their patients. This may reduce the number of noncompliant patients suffering from mental disorders that choose not to take their prescribed medications. As with any form of treatment, nutritional therapy should be supervised and doses should be adjusted as necessary to achieve optimal results.

## Abbreviations

ADD: attention deficit disorder

ADHD: attention deficit hyperactivity disorder

CSF: cerebrospinal fluid

DHA: docosahexaenoic acid

EPA: eicosapentaenoic acid

FDA: Food and Drug Administration

GABA: gamma-aminobutyric acid

OCD: obsessive-compulsive disorder

PPARs: peroxisomal proliferator-activated receptors

PTSD: post-traumatic stress disorder

SAM: S-adenosylmethionine

SSRI: selective serotonin reuptake inhibitors
